# Evidence for the Involvement of Fatty Acid Biosynthesis and Degradation in the Formation of Insect Sex Pheromone-Mimicking Chiloglottones in Sexually Deceptive *Chiloglottis* Orchids

**DOI:** 10.3389/fpls.2018.00839

**Published:** 2018-06-19

**Authors:** Darren C. J. Wong, Ranamalie Amarasinghe, Eran Pichersky, Rod Peakall

**Affiliations:** ^1^Ecology and Evolution, Research School of Biology, The Australian National University, Canberra, ACT, Australia; ^2^Department of Molecular, Cellular, and Developmental Biology, University of Michigan, Ann Arbor, MI, United States

**Keywords:** *Chiloglottis*, chiloglottone, sexual deception, pollination, transcriptome, fatty acid

## Abstract

Hundreds of orchid species secure pollination by sexually luring specific male insects as pollinators by chemical and morphological mimicry. Yet, the biochemical pathways involved in the synthesis of the insect sex pheromone-mimicking volatiles in these sexually deceptive plants remain poorly understood. Here, we explore the biochemical pathways linked to the chemical mimicry of female sex pheromones (chiloglottones) employed by the Australian sexually deceptive *Chiloglottis* orchids to lure their male pollinator. By strategically exploiting the transcriptomes of chiloglottone 1-producing *Chiloglottis trapeziformis* at distinct floral tissues and at key floral developmental stages, we identified two key transcriptional trends linked to the stage- and tissue-dependent distribution profiles of chiloglottone in the flower: (i) developmental upregulation of fatty acid biosynthesis and β-oxidation genes such as *KETOACYL-ACP SYNTHASE, FATTY ACYL-ACP THIOESTERASE*, and *ACYL-COA OXIDASE* during the transition from young to mature buds and flowers and (ii) the tissue-specific induction of fatty acid pathway genes in the callus (the insectiform odor-producing structure on the labellum of the flower) compared to the labellum remains (non-odor-producing) regardless of development stage of the flower. Enzyme inhibition experiments targeting KETOACYL-ACP SYNTHASE activity alone in three chiloglottone-producing species (*C. trapeziformis, C. valida*, and *C.* aff. *valida*) significantly inhibited chiloglottone biosynthesis up to 88.4% compared to the controls. These findings highlight the role of coordinated (developmental stage- and tissue-dependent) fatty acid gene expression and enzyme activities for chiloglottone production in *Chiloglottis* orchids.

## Introduction

Flowers have evolved diverse strategies to attract potential pollinators by conveying visual and olfactory signals (Raguso, 2004; Borghi et al., 2017). Some plants even use specific “semiochemical" signals that mimic the sex pheromone of female insects to sexually lure their highly specific male insect pollinators. This fascinating pollination strategy, commonly known as sexual deception (SD), has evolved multiple times on several continents (Gaskett, 2011). Amongst the Orchidaceace, hundreds of species spanning more than 20 genera across different subtribes are involved. Other cases are known in two other plant families (Bohman et al., 2016). In orchids, pollination by SD is often achieved when the male insect pollinator attempts to copulate with the flower, with volatiles holding the key to this interaction (Bohman et al., 2016; Wong et al., 2017b).

The Australian SD orchids of the genus *Chiloglottis* employ chiloglottones, a class of 2,5-dialkylcyclohexan-1,3-dione natural products (Peakall et al., 2010), to lure their specific male thynnine wasp pollinator (See Supplementary Figures 1A,B). For example, chiloglottone 1 is employed by *Chiloglottis trapeziformis* to attract its primary male pollinator, *Neozeleboria cryptoides* while the mostly allopatric *C. valida* employs chiloglottone 1 but attracts *N. monticola* for pollination. Conversely, a morphologically cryptic species with taxonomic affinity to *C. valida* (here after *C.* aff. *valida* secures pollination using a specific blend of chiloglottones 1 and 2 (Peakall et al., 2010; Peakall and Whitehead, 2014). In *C. trapeziformis*, chiloglottone 1 production has been shown to be specific to the densely clustered “insectiform” calli structure (callus) on the labellum, and its production is unexpectedly UV-B dependent (Falara et al., 2013). Flowers that open in the field become depleted of chiloglottone within 3–5 days when they are grown under light lacking in the UV-B range. Re-exposure to UV-B light, or natural sunlight, rapidly initiates chiloglottone production. Chiloglottone 1 is also dependent on the flowers’ developmental stage (Amarasinghe et al., 2015). In all but the very young buds, chiloglottone 1 can be detected when flowers are exposed to UV-B or sunlight, with levels increasing from trace amounts from manually opened young buds to appreciable amounts in very mature buds and in fully open flowers.

The formation of chiloglottones has been predicted to involve the condensation of intermediates of varying chain lengths in the fatty acid (FA) biosynthetic and degradative (β-oxidation) pathways, followed by decarbonylation (Franke et al., 2009; Bohman et al., 2016). For example, the condensation of activated 3-oxohexanoic acid (CoA or ACP) and activated 2-hexenoic acid precursors and subsequent decarboxylation may give rise to chiloglottone 1 (See Supplementary Figure 1C). As such, formation of chiloglottone 2 may involve activated 3-oxohexanoic acid (CoA or ACP) and activated 2-octenoic acid precursors. In plants, such intermediates are ubiquitous, existing in the plastids as ACP derivatives or the peroxisomes as CoA derivatives. However, just how these precursors are made available for chiloglottone formation in an otherwise iterative pathway is unknown. One hypothesis for this may involve the mid-cycle termination (i.e., after a specified number of cycles) of FA biosynthesis and/or degradation (β-oxidation) pathway enzymes (Bohman et al., 2016).

Previously, we reported that tissue-specific floral transcriptome analysis, comparing the chiloglottone-emitting callus with the non-active labellum of *C. trapeziformis*, revealed that FA biosynthesis and β-oxidation pathways were highly coordinated in a tissue-specific manner whereby pathway transcripts are often highly or exclusively expressed in the callus (Wong et al., 2017a). This finding matched with the known tissue-specific distribution of chiloglottone 1 (Falara et al., 2013; Amarasinghe et al., 2015) and supported the current hypothesis of chiloglottones formation (Franke et al., 2009; Bohman et al., 2016). Interestingly, no induction of FA pathways was observed under UV-B treatment in the callus of mature buds and flowers despite the activation of UVR8-mediated signaling pathways, suggesting an unknown mechanism operating on chiloglottone 1 production at the transcriptional and metabolic level.

Building upon these previous biochemical, chemical, and transcriptional observations linked to chiloglottone formation, here we address three outstanding questions using targeted gene expression analysis and molecular inhibitor experiments. We ask the following three specific questions: (1) Are FA metabolic pathway genes developmentally regulated during the transition from young buds to mature flowers in *C. trapeziformis*? (2) Are FA metabolic pathway genes regulated in a tissue-specific manner in *C. trapeziformis*? (3) Does the inhibition of FA biosynthesis impair chiloglottone production in a cross section of *Chiloglottis* orchids?

## Materials and Methods

### Study Species

Whole *Chiloglottis trapeziformis* plants with single flowers at two developmental stages, namely very young buds (*vyb*) and naturally opened flowers (*flw*) (Amarasinghe et al., 2015), were sampled from a colony growing naturally within the Australian National Botanic Gardens (Canberra, ACT, Australia) in September 2014. Whole *C. valida* and *C.* aff. *valida* plants with single naturally opened flowers were also sourced from wild populations within the Kosciuszko National Park in NSW, Australia, in November 2014. See supplementary materials for plant growth conditions.

### RNA Extraction, Library Construction, RNA Sequencing, and Transcriptome Analysis

Fifteen plants (three biological replicates with each replicate containing five individual plants) were used for each treatment. Floral tissues of *vyb* were carefully dissected to separate the stalked callus from the remaining labellum then immediately snap-frozen in liquid nitrogen. RNA extractions, library construction, and RNA sequencing was performed as previously described (Wong et al., 2017a; Xu et al., 2017). All raw sequence reads obtained in this study have been added to the existing BioProject accession PRJNA390683 and SRA study accession SRP109328^1^. Paired-end reads from (Wong et al., 2017a), and from non UV-B treated callus and labellum at very mature buds (*vmb*) and *flw* stages, were used in conjunction with sequenced tissue-specific reads at *vyb* stage obtained in this study and analyzed according to previously pipelines (Wong et al., 2017a). The classifications of bud and flower stages used in this study follows Amarasinghe et al. (2015). The *vyb* stage is characterized by a small and very tightly closed buds with both green stalk and bud. The *vmb* stage is characterized by larger buds that are about to open with sepals and petals beginning to separate. Fully open flowers define the *flw* stage. See supplementary materials for detailed methods.

### Pharmacological Inhibition Experiments and Chemical Analysis

All inhibition experiments were conducted on chiloglottone-depleted cut flowers from growth-chamber acclimatized whole plants (*C. trapeziformis, C. valida*, and *C.* aff. *valida*). Stock solutions (40 mM) of Cerulenin (Cayman Chemical, United States) were dissolved in Dimethyl Sulfoximine (Sigma, United States) and diluted to a final concentration of 100 μM with water (assay buffer). An assay buffer without the inhibitor served as the solvent control. The stalks of cut flowers were immersed into either 100 μM of Cerulenin (assay buffer) or solvent control. Next, the tip of each test tube was sealed with parafilm to ensure no direct contact between the solution and the flowers (3 flowers/tube) and then held in a test tube for 3 days. To induce chiloglottone production, UV-B treatments were conducted over a 2-h period on inhibitor-treated and control plants using a custom made light-box following Amarasinghe et al. (2015). The calli of all three species were immediately excised and assayed for chiloglottones as previously described (Falara et al., 2013). See supplementary materials for detailed chemical analysis methods.

### Statistical Analysis

Differential expression analysis was performed using DESeq2 (Love et al., 2014). Transcripts differentially expressed between any given contrasts are defined as having an absolute log2 fold change (log2FC) >0.5 with a false discovery rate (FDR) <0.05. For all inhibition experiments, at least six flowers were used in each treatment or control group. The outcomes of inhibition treatments were analyzed with Student’s *t*-test in R^2^.

## New Insights Into the Formation of Insect Sex Pheromone-Mimicking Chiloglottones

### Spatio-Temporal Gene Expression of Fatty Acid Pathways Coincides With Chiloglottone 1 Production in *C. trapeziformis*

In this study, we specifically addressed the first two objectives by interrogating the callus and labellum transcriptomes of *vyb* (obtained in this study; Supplementary Table 1) in conjunction with *vmb* and *flw* transcriptomes collected from the same colony in the same year obtained from our previous study (Wong et al., 2017a). Principal component analysis revealed that major differences among treatments was mainly driven by developmental stage followed by tissue specificity. (Supplementary Figure 2A). Enrichment (FDR <0.05) of gene ontology categories such as carbohydrate and lipid metabolic process were often observed in both developmental stage and tissue-specific contrasts (Supplementary Figures 2A–C). Our findings revealed that many genes of the fatty acid (FA) biosynthesis and β-oxidation pathways were only upregulated in the callus during the transition from *vyb* to *vmb*. This coincides with a strong enrichment of lipid metabolism processes (FDR < 6.43 × 10^-11^). Meanwhile, tissue-specific pairwise contrasts (callus vs. labellum) revealed that lipid metabolism process were also enriched in the callus regardless of developmental stage (FDR_*vmb*_ < 3.50 × 10^-6^, FDR_*vyb*_ < 1.81 × 10^-3^, FDR_*flw*_ < 2.02 × 10^-2^).

### Three Fatty Acid Biosynthesis and Two β-Oxidation Pathway Genes Exhibit Callus-Specific Developmental Upregulation

**FIGURE 1:**
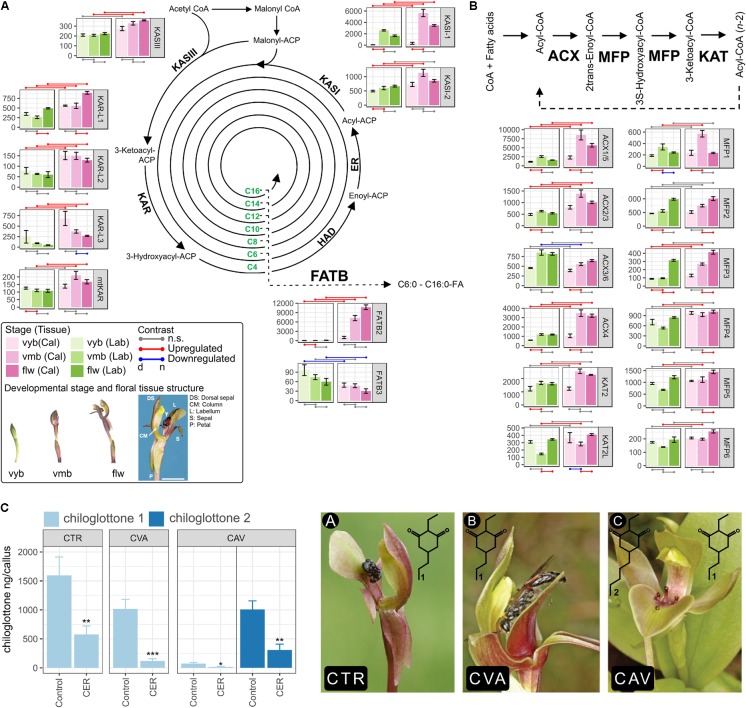
Fatty acid (FA) biosynthesis and degradation pathway gene expression in the callus and labellum tissues of *Chiloglottis trapeziformis* flowers. Differentially expressed genes of the FA **(A)** biosynthesis and **(B)** β-oxidation (degradation) are depicted. Values are normalized transcript counts ± SE. Shades of pink and green colors depict callus and labellum tissues at various flower developmental stage. Developmental stage and floral tissue structure of *C. trapeziformis* are depicted. The arrow indicates the callus tissue at the *flw* stage. Scale bar = 7 mm. Red and blue points connecting any two conditions depict statistically significant (FDR < 0.05, |log2FC| > 0.5) upregulation and downregulation, respectively. Gray points depict no significant differential expression. *n* and *d* represent the numerator and denominator conditions of each comparisons, respectively. **(C)** Chiloglottone amounts in the callus of *Chiloglottis* flowers following incubation with Cerulenin (CER) compared with controls following a 2-h UV-B exposure. Picture A, *C. trapeziformis* flower; Picture B, *C. valida* flower; Picture C, *C*. aff. *valida* flower. CTR, *C. trapeziformis*; CVA, *C*. *valida*; CAV, *C*. aff. *valida*. The number of flowers used in each treatment group are the following: CTR_CER_ = 10, CTR_Control_ = 9, CVA_CER_ = 12, CVA_Control_ = 8, CAV_CER_ = 6, CAV_Control_ = 7. Bars represent ± SE. Asterisks indicate significant differences between treatments at ^∗^*P* < 0.05, ^∗∗^*P* < 0.01, ^∗∗∗^*P* < 0.001 based on Student’s *t*-test. Light and dark blue colors indicate chiloglottone 1 and 2, respectively. All images have been reproduced with permission from the respective copyright holders. Please refer to the Section “Acknowledgments” for image credits.

Most of the FA biosynthesis such as one *KETOACYL-ACP SYNTHASE III* (*CtKASIII*), four *KETOACYL-ACP REDUCTASE* (*CtKAR-L1 – 3*, mitochondrial *CtKAR*), two *KETOACYL-ACP SYNTHASE I* (*CtKASI-1* and *CtKASI-2*), and one *FATTY ACYL-ACP THIOESTERASE* (*CtFATB2*) and FA β-oxidation pathway genes such as three *ACYL-COA OXIDASE* (*CtACX2/3, CtACX4*, and *CtACX1/5*) and one *MULTIFUNCTIONAL PROTEIN* (*CtMFP3*) were consistently upregulated in the callus compared to the labellum (**Figures 1A,B**). In addition, the transition from *vyb* to *vmb* stages generally involved coordinated upregulation of pathway genes, with transcripts remaining high in *flw* (no significant change from *vmb* to *flw*). Interestingly, four genes exhibited striking tissue (callus)-specific developmental upregulation (e.g., *CtKASI-*2, *CtFATB, CtACX2/3*, and *CtACX4*). These genes showed consistently (i) higher expression in the callus compared to the labellum and (ii) exhibited callus-specific developmental upregulation during the transition from *vyb* to *vmb*. We hypothesized that they may have direct involvement in chiloglottone 1 formation compared to those that are expressed at higher levels in the callus but are non-specifically upregulated in both callus and labellum tissues during the transitions from *vyb* to *vmb* (i.e., *CtKASI-1*, CtACX1*/*5, *CtMFP1, CtMFP2*, and *CtMFP3*) and *vmb* to *flw* (i.e., *CtKAR-L1, CtMFP3*, and *CtKAT2L*).

### Inhibition of Fatty Acid Biosynthesis Significantly Block Chiloglottone Production in *Chiloglottis* Flowers

In light of the striking developmental stage- and tissues-specific upregulation of several key FA pathway genes (**Figures 1A,B**), we hypothesized that inhibition of their activities may affect chiloglottone 1 biosynthesis in *C. trapeziformis*. Therefore, we tested the effect of KAS inhibition using Cerulenin. Studies in several plant species have shown that Cerulenin specifically inhibits KAS activity, and thus fatty acid elongation (Shimakata and Stumpf, 1982; Dehesh et al., 1998; Yasuno et al., 2004). We confirm that CtKASIs (CtKASI-2 and CtKASI-1) are potentially susceptible to Cerulenin inhibition as both protein sequence possesses the catalytic Cysteine-Histidine-Histidine triad active site (Wong et al., 2017a). Following a 2-h UV-B exposure of chiloglottone 1-depleted *C. trapeziformis* flowers, the amount of chiloglottone 1 in the calli treated with solvent control was 1,596 ± 317 ng/callus while 100 μM Cerulenin treatment significantly inhibited chiloglottone 1 production by 63.8% (*P* < 0.05) (**Figure 1C**). Motivated by these findings, we extended our test of the effect of Cerulenin on chiloglottone production to two other *Chiloglottis* species in a different clade to *C. trapeziformis*: *C. valida* that only produces chiloglottone 1 and *C.* aff. *valida* that produces chiloglottone 1 and chiloglottone 2. Following a 2-h UV-B treatment, the mean chiloglottone 1 levels in the controls of *C. valida* was 1,017 ± 165 ng/callus and the treatment significantly inhibited chiloglottone1 production by 88.4% (*P* < 0.001). Similarly, mean chiloglottone 1 and chiloglottone 2 levels in *C.* aff. *valida* controls were 73 ± 21 ng/callus and 1,007 ± 149 ng/callus, respectively. Cerulenin treatment significantly inhibited chiloglottone 1 and chiloglottone 2 production by 77.6% (*P* < 0.05) and 69.5% (*P* < 0.01), respectively (**Figure 1C**).

## Perspectives on the Biosynthesis of Insect Sex Pheromone-Mimicking Chiloglottones

Parallel temporal changes in enzyme activities, protein content, and their corresponding structural gene expression are often pivotal for developmentally regulated and/or tissue-specific volatile production in flowers (Dudareva et al., 2013). Confirmed cases where the biosynthesis of the semiochemicals involved in the sexual mimicry is known in SD orchids is presently limited to the biosynthesis of 7-, 9-, and 12-alkenes in *Ophrys* orchids (Schlüter et al., 2011; Xu et al., 2012; Sedeek et al., 2016) and (S)-β-citronellol in *Caladenia plicata* (Xu et al., 2017). In this study, a strategic developmental stage and tissue differential expression analysis of *C. trapeziformis* floral transcriptomes identified two key transcriptional trends linked to the distribution of chiloglottone in the flowers: (i) During the transition from *vyb* to *vmb*, large suites of FA biosynthesis and β-oxidation genes are upregulated. (ii) In all development stages tested (especially *vmb*), FA pathway genes were consistently induced in the callus compared to the labellum (**Figure 1**). Despite this strong coordinated regulation, no genes from other major biosynthetic pathway (e.g., glycolysis and tricarboxylic acid) showed similar preferential expression (Supplementary Tables 2, 3). Our findings lend further support to the existing hypothesis of chiloglottone formation *in planta* via a FA biosynthetic route and demonstrate a key role of floral developmental transitions (i.e., *vyb* to *vmb*) in the priming of FA pathway gene expression to initiate chiloglottone biosynthesis.

Based on tissue-specific differences in *vmb* and *flw*, we have previously summarized the potential roles of several prioritized FA pathway steps in chiloglottone 1 biosynthesis (Wong et al., 2017a). Considering the new developmental stage contrasts obtained in this study, here we identified a smaller subset of genes (i.e., *CtKASI-2, CtFATB2, CtACX2/3*, and CtACX4) that may have direct implications for chiloglottone 1 formation (**Table 1**). Of particular interests is the KASI paralog, *CtKASI-2.* We predict that CtKASI-2 may be implicated in both precursor supply (i.e., 3-ketohexanyl-ACP) and directly in the formation of chiloglottone 1 through the condensation of 3-ketohexanyl-ACP and 2-hexenyl-CoA.

**Table 1:** Potential roles of key fatty acid biosynthesis and β-oxidation pathway genes implicated in chiloglottone 1 biosynthesis prioritized in this study.

Transcript	Enzyme	Pathway	Role	Supporting evidence	Reference
CtACX2/3 and CtACX4	ACX	β-oxidation	Precursor supply (e.g., 2-hexenyl-CoA)	*Arabidopsis* ACXs possess medium-to-long (AtACX2, C14:0 – C20:0; AtACX3, C8:0 – C14:0) and short-to-medium (AtACX4, C4:0 to C8:0) chain substrate specificities. CtACX2/3 and CtACX4 may facilitate 2-hexenyl-CoA production via a continuous passage through the β-oxidation spiral.	(Reviewed in Li-Beisson et al., 2013)
CtKASI-2^†^	KASI	Biosynthesis	Precursor supply (e.g., 3-ketohexanyl-ACP)	Paralogs of KASI in short FA-accumulating plants (e.g., coconuts) possesses additional short chain length Acyl-ACP substrate specificities (e.g., C4:0). CtKASI-2 may possess the latter preference and facilitate 3-ketohexanyl-ACP production.	Yuan et al., 2015
CtFATB2^†^	FATB	Biosynthesis	Mid-cycle termination	Tissue (fruit)-specific FATB paralogs in short FA-accumulating species (e.g., oil palm and coconut fruits) possesses short-to-medium chain (C8:0-C14:0) acyl-ACP substrate preference. CtFATB2 may compete between acyl chain elongation and premature cleavage of acyl-ACP.	Jing et al., 2011; Dussert et al., 2013
CtKASI-2^†^	KASI	Biosynthesis	Condensation	Condensation of activated β-ketoacyl starter with α,β-unsaturated-acyl substrate to form various 2,5-dialkylcyclohexane-1,3-diones in bacteria. CtKASI-2 may be responsible for the condensation of 3-ketohexanyl-ACP and 2-hexenyl-CoA to form 2-ethyl-5-propylcyclohexan-1,3-dion-4-carboxylate, the penultimate precursor to chiloglottone 1.	Fuchs et al., 2013; Mori et al., 2016

*^†^Paralogs unique to C. trapeziformis*.

To ascertain the role of KASI in chiloglottone biosynthesis, we performed KASI inhibition experiments using Cerulenin. Cerulenin, irreversibly inhibits KASI by forming a covalent bond with the cysteine active site (Moche et al., 1999; Johansson et al., 2008). We show that Cerulenin consistently inhibited chiloglottone production in the callus of three *Chiloglottis* species (i.e., *C. trapeziformis, C. valida, C.* aff. *valida*) up to 88.4% upon induction with UV-B compared to controls. (**Figure 1C**). Our findings provide the first biochemical evidence supporting FA biosynthesis as the major biosynthetic route for chiloglottones. These findings also indicate that chiloglottone induction by UV-B, tissue-specificity (Falara et al., 2013; Amarasinghe et al., 2015), and molecular pathways (Wong et al., 2017a) involved in chiloglottone formation are potentially conserved across *Chiloglottis*. To ascertain whether co-ordinately regulated patterns of FA pathways linked to chiloglottones in *C. trapeziformis* are also relevant in *C. valida* and *C.* aff. *valida*, systems-based comparative approaches can be adopted (Schilmiller et al., 2012; Wong and Matus, 2017). This could include strategic developmental stage- and tissue-specific differential expression as well as integrated network analysis of metabolites and genes (Wong et al., 2017b).

While in plants there is no evidence that Cerulenin directly/indirectly inhibits FA β-oxidation (Shimakata and Stumpf, 1982; Dehesh et al., 1998; Yasuno et al., 2004), we cannot yet rule out the possibility that FA β-oxidation as an alternative route for chiloglottone biosynthesis (Bohman et al., 2016). This is because any inhibition of KAS enzymes by Cerulenin may have an indirect effect – reducing the levels of *de novo* synthesized FAs (e.g., C16/C18) – and thus restricting further catabolism (via β-oxidation) to yield alternative activated (CoA) FA precursors of appropriate chain lengths for chiloglottone biosynthesis. Transcriptome observations showing coordinated (developmental stage- and tissue-dependent) expression of several *ACX* in *C. trapeziformis* also support the possibility that one or both putative chiloglottone precursors could be obtained by FA β-oxidation. Nonetheless, our results reinforce the current hypothesis that FA biosynthesis serves as the crucial “starting point” for chiloglottones formation. Future studies will require targeted knockdown of pathway candidates and metabolomics analysis for activated (ACP/CoA) FA precursors to ascertain whether chiloglottone production is largely determined by precursor availability (via the FA biosynthesis and/or β-oxidation pathway) or during the condensation of activated precursors that may involve novel KAS activities. Together, these new findings highlight the role of coordinated (developmental stage- and tissue-dependent) FA gene expression and enzyme activities for chiloglottone production in our study species and may have widespread implications for *Chiloglottis* and other orchid genera employing chiloglottones for SD pollination (Peakall et al., 2010).

## Author Contributions

DW and RA performed the experiments and analyzed the data. RP and EP secured funding, designed the study, and coordinated the experiments and data analysis. DW wrote the article with assistance from EP and RP. All authors have read and approved the paper.

## Conflict of Interest Statement

The authors declare that the research was conducted in the absence of any commercial or financial relationships that could be construed as a potential conflict of interest.

## Footnotes

1 http://www.ncbi.nlm.nih.gov/sra

2 https://www.r-project.org/
