# Supplementary material for: Evidence for the Involvement of Fatty Acid Biosynthesis and Degradation in the Formation of Insect Sex Pheromone-Mimicking Chiloglottones in Sexually Deceptive Chiloglottis Orchids
Source: Front Plant Sci. 2018 Jun 19;9:839. doi: 10.3389/fpls.2018.00839 (PMC6018206; doi:10.3389/fpls.2018.00839)
Supplement: Supplementary file 1 [file Presentation_1.PDF]

Supplementary material

Evidence for the involvement of fatty acid biosynthesis and degradation in the formation of insect sex pheromone-mimicking chiloglottones in sexually deceptive *Chiloglottis* orchids

Darren C.J. Wong[1†], Ranamalie Amarasinghe[1], Eran Pichersky[2], and Rod Peakall[1]

[1]Ecology and Evolution, Research School of Biology, The Australian National University, Acton ACT 2601, Australia [2]Department of Molecular, Cellular, and Developmental Biology, University of Michigan, Ann Arbor, Michigan 48109, USA.

[†]**Correspondence:**
Darren CJ Wong
darren.wong@anu.edu.au
+61 2 6125 3059

Supplementary figures and tables

Supplementary table 1. Summary of RNA sequencing analysis metrics obtained in this study. All raw sequence reads have been added to existing BioProject accession PRJNA390683 and SRA study accession SRP109328 (http://www.ncbi.nlm.nih.gov/sra).

Sample ID	Tissue	Developmental Stage	Treatment	Raw reads†	Trimmed reads†	Total alignments† (unique)	Total alignments† (Multi-mapped)	NCBI Biosample ID
CT000009	Cal	vyb	Cov	17.1	13.2	12.8	60.0	SAMN08817952
CT000010	Lab	vyb	Cov	20.1	14.9	14.4	65.0	SAMN08817953
CT000021	Cal	vyb	Cov	9.3	6.8	6.5	30.7	SAMN08817956
CT000022	Lab	vyb	Cov	20.0	14.2	13.7	61.4	SAMN08817957
CT000023	Cal	vyb	Cov	14.1	11.4	11.0	49.4	SAMN08817965
CT000024	Lab	vyb	Cov	16.5	12.9	12.4	55.4	SAMN08817966

vyb, very young buds; Cov, covered; Cal, callus; Lab, labellum; MM, multi-mapped; † million.

Supplementary table 2. Summary of glycolysis pathway gene expression changes in the callus during the transition from *vyb* to *vmb* (D1) and *vmb* to *flw* (D2) and in the labellum during the transition from *vyb* to *vmb* (D4) and *vmb* to *flw* (D5). Tissue-specific differential expression between the callus vs labellum in *vyb*, *vmb*, and *flw* are shown for TS1, TS2, and the TS3 contrast, respectively. Values depict fold change (log2) between respective contrasts. Asterisks depict statistically significant (FDR < 0.05, |log2FC| > 0.5) upregulation or downregulation.

Pathway	Protein	Transcript	D1	D2	D4	D5	TS1	TS2	TS3	D1	D2	D4	D5	TS1	TS2	TS3
Glycolysis (Central)	Hexokinase	CHILtrap-81875.0	-1.82	-0.38	-1.16	0.4	-0.24	-0.9	-1.68	*		*			*	*
		CHILtrap-81875.1	0.63	-1.03	0.16	-0.29	-0.68	-0.21	-0.96							
	Glucose-6-phosphate isomerase	CHILtrap-98369.0	-0.56	0.76	-0.38	0.83	0.75	0.57	0.5	*	*		*	*	*	*
	Phosphofructokinase	CHILtrap-71387.0	-0.91	0.3	0.25	-0.05	0.3	-0.86	-0.51	*					*	
		CHILtrap-86536.0	0.22	0.03	-0.35	0.36	0.17	0.73	0.4						*	
		CHILtrap-93620.0	-0.88	0.22	-2.19	1.56	-0.23	1.08	-0.26							
	Aldolase	CHILtrap-69902.0	0.04	0.03	0.32	0.35	0.48	0.21	-0.11							
		CHILtrap-73555.0	-3.89	-0.81	-3.74	0.15	-0.11	-0.26	-1.22	*		*				
	GAPDH	CHILtrap-107168.1	1.72	0.13	2.11	-1.14	-0.2	-0.58	0.69	*		*				
		CHILtrap-89004.0	1.18	-0.25	-0.28	0.4	-1.05	0.41	-0.24	*				*		
	Phosphoglycerate	CHILtrap-91998.0	-0.62	0.45	-0.93	0.64	0.06	0.37	0.18							
	mutase	CHILtrap-98494.0	0.71	-0.04	0.9	-0.25	0.21	0.02	0.23	*		*				
		CHILtrap-99002.0	-0.74	0.61	-0.14	0.3	0.3	-0.3	0	*						
		CHILtrap-99028.0	0.22	-0.23	0.55	-0.29	0.41	0.08	0.14			*				
	Enolase	CHILtrap-86373.0	-0.03	0.47	0.04	0.51	0.28	0.21	0.16				*			
		CHILtrap-97768.0	-1.33	0.69	-0.62	0.25	0.09	-0.62	-0.18	*	*	*			*	
	Pyruvate kinase	CHILtrap-112711.0	-0.33	0.13	-0.03	0.32	0.03	-0.27	-0.45							
		CHILtrap-69695.0	0.34	-0.38	0.75	-0.44	0.55	0.13	0.19			*				
		CHILtrap-86202.0	-0.61	0.15	-0.13	0.06	0.4	-0.08	0.01	*						
		CHILtrap-86970.0	-0.17	0.34	-0.02	0.41	0.34	0.18	0.11							
		CHILtrap-87959.0	0.49	0.23	0.07	0.56	0.08	0.49	0.16				*			

Supplementary table 3. Summary of tricarboxylic acid pathway gene expression changes in the callus during the transition from *vyb* to *vmb* (D1) and *vmb* to *flw* (D2) and in the labellum during the transition from *vyb* to *vmb* (D4) and *vmb* to *flw* (D5). Tissue-specific differential expression between the callus vs labellum in *vyb*, *vmb*, and *flw* are shown for TS1, TS2, and the TS3 contrast, respectively. Values depict fold change (log2) between respective contrasts. Asterisks depict statistically significant (FDR < 0.05, |log2FC| > 0.5) upregulation or downregulation.

Pathway	Protein	Transcript	D1	D2	D4	D5	TS1	TS2	TS3	D1	D2	D4	D5	TS1	TS2	TS3
TCA (Central)	Aconitase	CHILtrap-75079.0	-0.5	0.13	-0.5	0.22	0.12	0.12	0.03							
		CHILtrap-87250.0	0.12	0.26	0	0.41	0.31	0.43	0.28							
	Isocitrate dehydrogenase	CHILtrap-74459.0	-1.02	1.56	-0.57	0.42	-0.15	-0.61	0.54	*	*					
		CHILtrap-83683.0	-0.63	0.18	-0.42	0.12	0.16	-0.05	0.02	*						
		CHILtrap-95499.0	-0.91	1.07	-1.5	0.29	-0.66	-0.06	0.71			*				
		CHILtrap-77069.0	0.04	-0.32	0.16	0	0.26	0.14	-0.18							
	2-oxoglutarate dehydrogenase	CHILtrap-108658.2	0.36	0.1	0.2	0.18	0.04	0.21	0.12							
		CHILtrap-113731.0	1.17	-1.18	1.35	-0.38	0.1	-0.07	-0.87	*	*	*				
		CHILtrap-113731.2	0.14	0.08	0.37	-0.14	-0.11	-0.34	-0.11							
		CHILtrap-113731.3	0.98	-0.04	1.05	-0.4	0.09	0.02	0.38	*		*				
		CHILtrap-113731.7	-0.04	0.15	0.28	0.12	0.33	0.02	0.05							
		CHILtrap-61833.0	-0.08	0.47	-0.35	0.51	0.12	0.39	0.35				*			
	Succinyl-CoA ligase	CHILtrap-100806.0	-0.08	0.63	-0.15	0.67	0.25	0.32	0.29		*		*			
		CHILtrap-91361.0	-0.37	0.58	-0.55	0.93	0.21	0.4	0.05		*	*	*			
	Succinate dehydrogenase	CHILtrap-85849.0	0.26	-0.17	0.09	-0.27	0.11	0.27	0.37							
	Fumarase	CHILtrap-64473.0	-0.3	0.85	-0.35	0.19	-0.23	-0.18	0.48		*					
	Malate dehydrogenase	CHILtrap-97014.0	0.03	0.24	0.33	0.12	0.29	-0.02	0.1							
		CHILtrap-92766.0	-0.59	0.6	0.08	0.87	0.53	-0.15	-0.42	*	*		*	*		
		CHILtrap-89361.0	-0.03	0.1	0.15	0.05	0.23	0.04	0.1							

Supplementary Figure 1. The diversity, distribution, and biosynthesis of chiloglottones. **(A)** Chemical structure of the six known biologically active chiloglottones produced by *Chiloglottis* orchids (Franke et al., 2009). 1, 2-ethyl-5-propylcyclohexane-1,3-dione; 2, 2-ethyl-5-pentylcyclohexane-1,3-dione; 3, 2-butyl-5-methylcyclohexane-1,3-dione; 4, 5-allyl-2-ethylcyclohexane-1,3-dione; 5, 2-butyl-5-propylcyclohexane-1,3-dione; 6, 2-hexyl-5-methylcyclohexane-1,3-dione. **(B)** The distribution of biologically active chiloglottones identified in various *Chiloglottis* orchids according to (Peakall et al., 2010) **(C)** Predicted biosynthetic steps for chiloglottone using chiloglottone 1 as an illustration. Purple and red compounds indicate activated (X – CoA/ACP) precursors. Biosynthesis of chiloglottones may involve the decarbonylation of respective carboxylate intermediate, of which the latter is potentially formed via the cyclization of activated precursors (of different chain lengths) (Bohman et al., 2016; Franke et al., 2009).

A

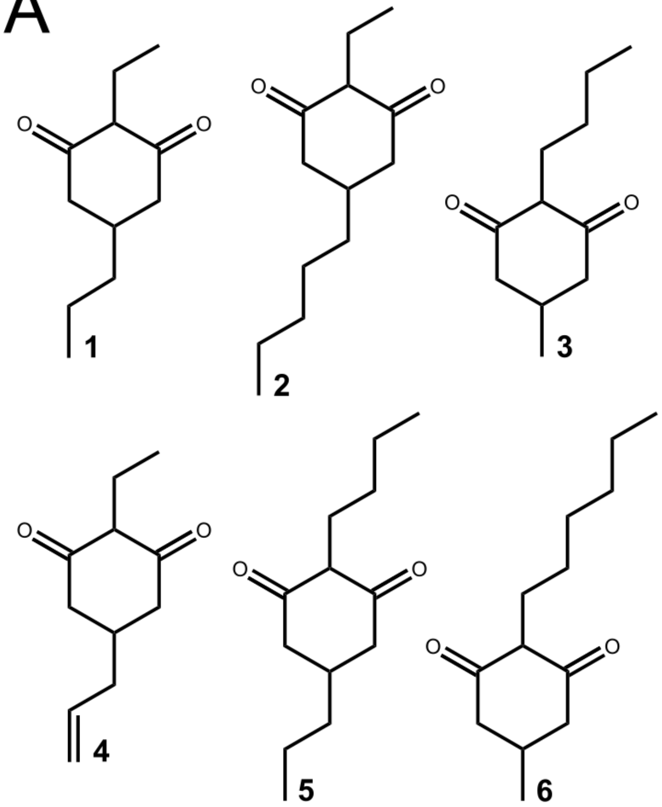

B

	chiloglottones					
	1	2	3	4	5	6
C. trapeziformis	Y					
C. valida	Y					
C. seminuda	Y					
C. aff. *pluricallata 1*	Y					
C.* aff. *valida 1	Y	Y				
C. platyptera	Y	Y				
C. pluricallata	Y	Y				
C. chlorantha			Y			
C. aff. *jeanesii*			Y			
C. jeanesii			Y			
C. triceratops				Y		
C. sp *(bifaria)*				Y		
C. turfosa				Y		
C. trilabra	Y		Y			
C. gunnii		Y				
C. grammata		Y				
C. formicifera	Y			Y		
C. sphyrnoides					Y	

C

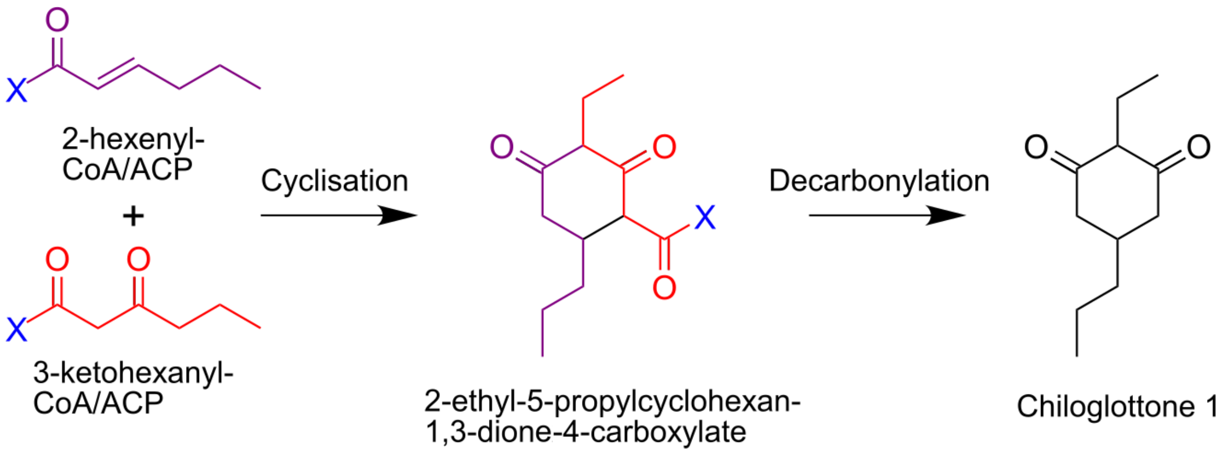

Supplementary Figure 2. Analysis of tissue-specific *C. trapeziformis* transcriptome during floral development. **(A)** Transcriptomes of *very young buds* (*vyb*) (obtained in this study) were analysed alongside *very mature buds* (*vmb*) and *flower* (*flw*) transcriptomes of (Wong et al., 2017). The first two axes of the principal component analysis for the final set of 24,838 expressed transcripts showed a clear separation of the floral transcriptome based on developmental stage (young vs mature stages in PC1: i.e. *vyb* and vmb/*flw*) followed by tissue specificity (callus vs labellum in PC2) . **(B)** Summary of differentially expressed genes in the callus during the transition from *vyb* to *vmb* (D1) and *vmb* to *flw* (D2) and in the labellum during the transition from *vyb* to *vmb* (D4) and *vmb* to *flw* (D5). Tissue-specific differential expression between the callus vs labellum in *vyb*, *vmb*, and *flw* are TS1, TS2, TS3 contrast, respectively. The number of upregulated and downregulated genes in each comparison are shown. **(C)** Summary of enriched gene ontology (GO) SLIM categories (FDR < 0.05) describing high-level plant biological processes for upregulated (red) and downregulated (blue) genes in each comparisons identified in **(B)**. Circle size depicts the number of genes (log2) annotated in each enriched category and its opacity (enrichment score) depicts enrichments ($-\log10_{FDR}$) for each enriched category. Asterisks in red highlight lipid metabolism process (GO:0006629; predominantly fatty acid (FA) biosynthesis and β-oxidation pathway genes) that were found to be highly enriched in upregulated genes during the floral development transitions (i.e. *vyb* to *vmb*) and between specific floral tissues regardless of developmental stage.

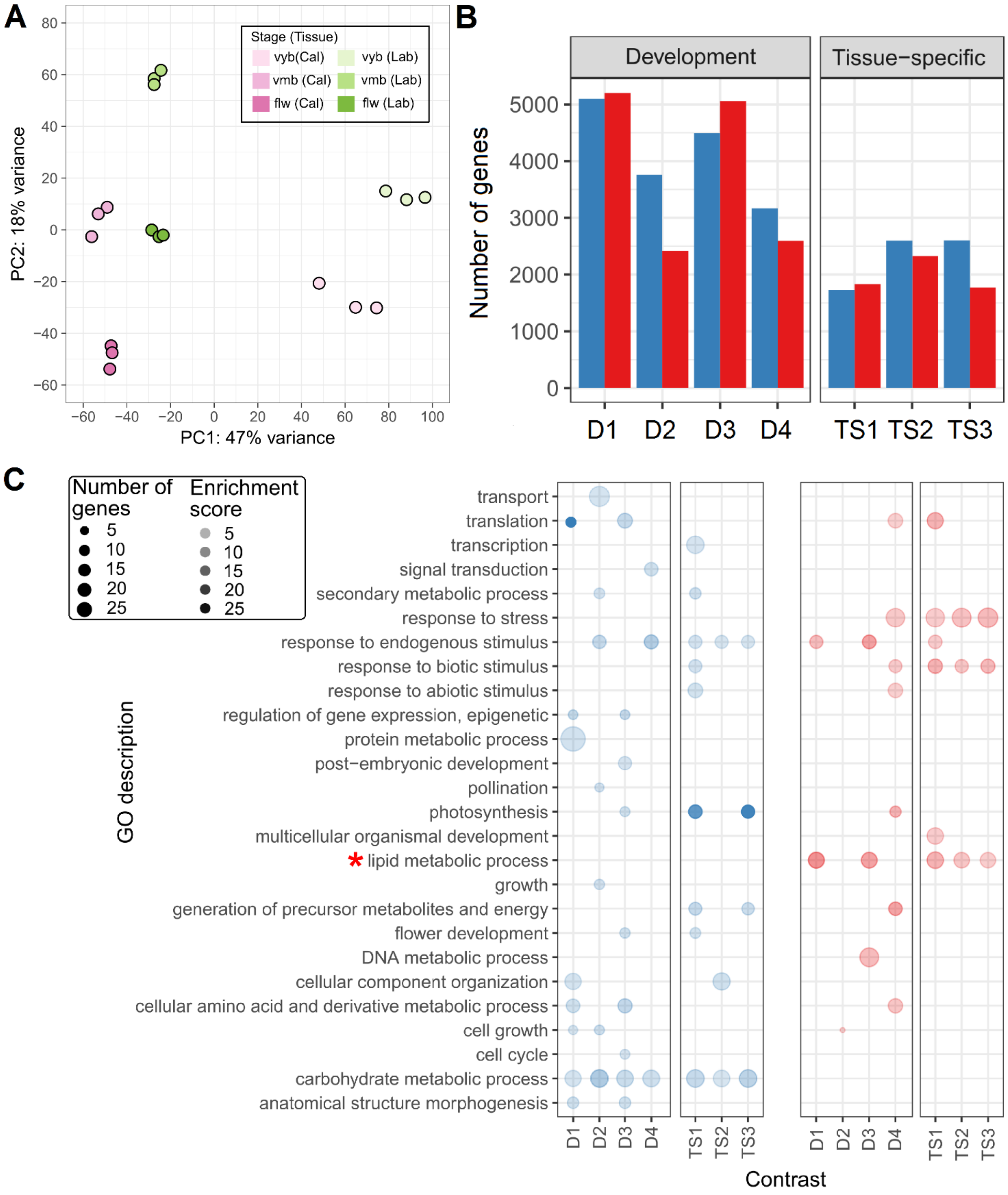
A
Stage (Tissue)
vyb(Cal)
vmb (Cal)
flw (Cal)
vyb (Lab)
vmb (Lab)
flw (Lab)
PC2: 18% variance
PC1: 47% variance
80
60
40
20
0
−20
−40
−60
−60
−40
−20
0
20
40
60
80
100
B
Development
Tissue−specific
Number of genes
5000
4000
3000
2000
1000
0
D1
D2
D3
D4
TS1
TS2
TS3
C
Number of genes
5
10
15
20
25
Enrichment score
5
10
15
20
25
GO description
transport
translation
transcription
signal transduction
secondary metabolic process
response to stress
response to endogenous stimulus
response to biotic stimulus
response to abiotic stimulus
regulation of gene expression, epigenetic
protein metabolic process
post−embryonic development
pollination
photosynthesis
multicellular organismal development
* lipid metabolic process
growth
generation of precursor metabolites and energy
flower development
DNA metabolic process
cellular component organization
cellular amino acid and derivative metabolic process
cell growth
cell cycle
carbohydrate metabolic process
anatomical structure morphogenesis
D1
D2
D3
D4
TS1
TS2
TS3
D1
D2
D3
D4
TS1
TS2
TS3
Contrast

Detailed methods

Plant growth conditions

Plants were kept in a growth chamber under these conditions: a minimum acclimatisation period of 5 days, a day/night cycle of 12 hours, temperatures of 20°C (day) and 15°C (night), and white light (300 µmol m^{-2} s^{-1}) lacking in the UV spectrum (< 400 nm).

RNA extraction, library construction, and RNA sequencing

Approximately, 100mg of ground tissues was used to obtain the total RNA using the Qiagen® RNeasy ® plant mini kit (Qiagen, Australia). Isolation of mRNA was conducted with NEBNext Poly(A) mRNA Magnetic Isolation Module and library construction using the Ultra RNA Library Prep Kit for Illumina (NEB, Australia), according to the manufacturer's protocols. All libraries were sequenced on the Illumina HiSeq 2500 platform at the ANU.

RNA sequencing data analysis

Trimming and quality filtering of raw paired-end (150 bp) reads were performed with Trimmomatic v0.35 (Bolger et al., 2014). *De novo* transcriptome re-assembly was performed using Trinity v2.1.1 (Grabherr et al., 2011) with non-redundant reads obtained in this study and from an earlier study (Wong et al., 2017). Bowtie v1.0.1 (Langmead et al., 2009) was used to multi-map reads to the newly assembled transcriptome and count summarization of transcripts was performed using Corset v1.04 (Davidson and Oshlack, 2014). Transcript abundance are expressed as normalized count values and lowly-expressed transcripts were filtered prior to differential expression (DE) analysis. Differentially expression analysis was performed using DESeq2 (Love et al., 2014). Transcripts differentially expressed between any given contrasts used in this study are defined as having an absolute log2 fold change (log2FC) > 0.5 with a false discovery rate (FDR) < 0.05. Enriched (FDR < 0.05) gene ontology (GO) SLIM categories were identified using BINGO (Maere et al., 2005). All raw sequence reads have been added to the existing BioProject accession PRJNA390683 and SRA study accession SRP109328 (http://www.ncbi.nlm.nih.gov/sra).

Chemical analysis

Excised calli were washed for 3 mins in 100 µl of HPLC grade Dichloromethane (DCM) spiked with 5-methyl-1,3-cyclohexanedione (Aldrich CAS 4341-24-6) at 20 ng/µl, as an internal standard. Gas chromatographic with mass spectrometry (GC-MS) analysis of each sample was conducted on an Agilent Technologies 6890N GC coupled with a 5973 Mass Selective Detector (Agilent Technologies, USA) equipped with a SGE BP21 column (30 m x 0.25 mm x 0.25 µm) connected directly to the MS detector. For each sample, 4 µl of extract was injected splitless into the inlet at 250° C, the column was held at 40° C for 1 min, then programmed at 10° C per min to 230° C and held for 15 mins. Helium served as the carrier gas at a flow of 2 ml/min. Quantitation was performed using the Agilent Technologies Chemstation software based on corrected percent areas relative to the internal standard.

References

Bohman, B., Flematti, G. R., Barrow, R. A., Pichersky, E., and Peakall, R. (2016). Pollination by sexual deception - it takes chemistry to work. *Curr. Opin. Plant Biol.* 32, 37–46.

doi:10.1016/j.pbi.2016.06.004.

Bolger, A. M., Lohse, M., and Usadel, B. (2014). Trimmomatic: A flexible trimmer for Illumina sequence data. *Bioinformatics* 30, 2114–2120. doi:10.1093/bioinformatics/btu170.

Davidson, N. M., and Oshlack, A. (2014). Corset: enabling differential gene expression analysis for de novo assembled transcriptomes. *Genome Biol.* 15, 410. doi:10.1186/s13059-014-0410-6.

Franke, S., Ibarra, F., Schulz, C. M., Twele, R., Poldy, J., Barrow, R. A., et al. (2009). The discovery of 2,5-dialkylcyclohexan-1,3-diones as a new class of natural products. *Proc. Natl. Acad. Sci.* 106, 8877–82. doi:10.1073/pnas.0900646106.

Grabherr, M. G., Haas, B. J., Yassour, M., Levin, J. Z., Thompson, D. a, Amit, I., et al. (2011). Full-length transcriptome assembly from RNA-Seq data without a reference genome. *Nat. Biotechnol.* 29, 644–652. doi:10.1038/nbt.1883.

Langmead, B., Trapnell, C., Pop, M., and Salzberg, S. (2009). Ultrafast and memory-efficient alignment of short DNA sequences to the human genome. *Genome Biol.* 10, R25. doi:10.1186/gb-2009-10-3-r25.

Love, M. I., Huber, W., and Anders, S. (2014). Moderated estimation of fold change and dispersion for RNA-seq data with DESeq2. *Genome Biol.* 15, 550. doi:10.1186/s13059-014-0550-8.

Maere, S., Heymans, K., and Kuiper, M. (2005). BiNGO: A Cytoscape plugin to assess overrepresentation of Gene Ontology categories in Biological Networks. *Bioinformatics* 21, 3448–3449. doi:10.1093/bioinformatics/bti551.

Peakall, R., Ebert, D., Poldy, J., Barrow, R. A., Francke, W., Bower, C. C., et al. (2010). Pollinator specificity, floral odour chemistry and the phylogeny of Australian sexually deceptive Chiloglottis orchids: Implications for pollinator-driven speciation. *New Phytol.* 188, 437–450. doi:10.1111/j.1469-8137.2010.03308.x.

Wong, D. C. J., Amarasinghe, R., Rodriguez-delgado, C., Eyles, R., Pichersky, E., and Peakall, R. (2017). Tissue-Specific Floral Transcriptome Analysis of the Sexually Deceptive Orchid Chiloglottis trapeziformis Provides Insights into the Biosynthesis and Regulation of Its Unique UV-B Dependent Floral Volatile , Chiloglottone 1. *Front. Plant Sci.* 8, 1260. doi:10.3389/fpls.2017.01260.
